# Immunological Evidence of Variation in Exposure and Immune Response to *Bacillus anthracis* in Herbivores of Kruger and Etosha National Parks

**DOI:** 10.3389/fimmu.2022.814031

**Published:** 2022-02-14

**Authors:** Sunday O. Ochai, Jan E. Crafford, Ayesha Hassim, Charles Byaruhanga, Yen-Hua Huang, Axel Hartmann, Edgar H. Dekker, O. Louis van Schalkwyk, Pauline L. Kamath, Wendy C. Turner, Henriette van Heerden

**Affiliations:** ^1^ Department of Veterinary Tropical Diseases, Faculty of Veterinary Science, University of Pretoria, Pretoria, South Africa; ^2^ Wisconsin Cooperative Wildlife Research Unit, Department of Forest and Wildlife Ecology, University of Wisconsin-Madison, Madison, WI, United States; ^3^ Etosha Ecological Institute, Ministry of Environment, Forestry and Tourism, Okaukuejo, Namibia; ^4^ Office of the State Veterinarian, Department of Agriculture, Forestry and Fisheries, Government of South Africa, Skukuza, South Africa; ^5^ Department of Migration, Max Planck Institute of Animal Behavior, Radolfzell, Germany; ^6^ School of Food and Agriculture, University of Maine, Orono, ME, United States; ^7^ U.S. Geological Survey, Wisconsin Cooperative Wildlife Research Unit, Department of Forest and Wildlife Ecology, University of Wisconsin-Madison, Madison, WI, United States

**Keywords:** Anthrax, enzyme-linked immunosorbent assay (ELISA), *Equus quagga*, passive disease surveillance, serology, toxin neutralization assay (TNA), *Tragelaphus strepsiceros*, adaptive immunity

## Abstract

Exposure and immunity to generalist pathogens differ among host species and vary across spatial scales. Anthrax, caused by a multi-host bacterial pathogen, *Bacillus anthracis*, is enzootic in Kruger National Park (KNP), South Africa and Etosha National Park (ENP), Namibia. These parks share many of the same potential host species, yet the main anthrax host in one (greater kudu (*Tragelaphus strepsiceros*) in KNP and plains zebra (*Equus quagga*) in ENP) is only a minor host in the other. We investigated species and spatial patterns in anthrax mortalities, *B. anthracis* exposure, and the ability to neutralize the anthrax lethal toxin to determine if observed host mortality differences between locations could be attributed to population-level variation in pathogen exposure and/or immune response. Using serum collected from zebra and kudu in high and low incidence areas of each park (18- 20 samples/species/area), we estimated pathogen exposure from anti-protective antigen (PA) antibody response using enzyme-linked immunosorbent assay (ELISA) and lethal toxin neutralization with a toxin neutralization assay (TNA). Serological evidence of pathogen exposure followed mortality patterns within each system (kudus: 95% positive in KNP versus 40% in ENP; zebras: 83% positive in ENP versus 63% in KNP). Animals in the high-incidence area of KNP had higher anti-PA responses than those in the low-incidence area, but there were no significant differences in exposure by area within ENP. Toxin neutralizing ability was higher for host populations with lower exposure prevalence, i.e., higher in ENP kudus and KNP zebras than their conspecifics in the other park. These results indicate that host species differ in their exposure to and adaptive immunity against *B. anthracis* in the two parks. These patterns may be due to environmental differences such as vegetation, rainfall patterns, landscape or forage availability between these systems and their interplay with host behavior (foraging or other risky behaviors), resulting in differences in exposure frequency and dose, and hence immune response.

## Introduction

Disease dynamics may be shaped by the spatial structure of host-pathogen encounter rates, and how the frequency or dose of pathogen exposure affects host susceptibility and immunity to infection (1). Generalist pathogens can infect multiple host species and differ in their infection intensity or severity across hosts, and many previous studies have strived to understand the risk of infection among different host species (2, 3). There is an abundance of knowledge on how multi-host pathogens evolve and how host species differ in their susceptibility and immune responses (2, 4–6), both spatially and within a particular environment, but there is little information on within-species variation in exposure and immune responses. It is therefore imperative to study within-species differences in exposure and immunity among populations for a better understanding of both disease progression as well as between host transmission dynamics.

Anthrax, an archetypal multi-host disease, is a zoonosis that affects a wide range of species, although its most susceptible hosts are mammalian herbivores. Anthrax is caused by the gram-positive, capsule- and endospore-forming *Bacillus anthracis* bacterium. This pathogen must kill its animal host in a bid to further spread. Disease progression typically occurs either as acute or peracute septicaemia following incubation of 2-8 days (7). The variation in the incubation period could be due to the size of the infectious dose encountered and/or the exposure intervals (7–9). After the death of the host, blood oozes from the body orifices, exposing vegetative cells to oxygen, which triggers sporulation. The resulting endospores can survive in the soil for years until uptake (normally ingestion) by another susceptible host, within which the spores cross the epithelium and can germinate forming vegetative cells. This germination, followed by further propagation and an increase in cells producing toxins (10, 11), ultimately leads to the death of the host (12). Due to the acute and peracute nature of anthrax, diagnosis is mainly based on detection of the pathogen post-mortem through molecular identification, microscopy and culture (13–15). The detection of specific antibodies in serum from live animals can, however, provide information on previous exposure to the pathogen.

For the development of immunity against anthrax, the host must be able to resist the establishment of disease or stall its progression (16). The virulence factors of *B. anthracis* are encoded on two plasmids namely pXO1, which is responsible for the production of the toxins, and pXO2, which codes for the poly-ɣ-D-glutamic acid capsule that helps the pathogen avoid detection by the host immune system (17, 18). The pXO1 plasmid encodes for the cell-binding protective antigen protein (PA), and two enzymes, the lethal factor (LF) and the oedema factor (EF) proteins. PA can combine with either LF or EF to form lethal toxin (LT) or oedema toxin (ET) respectively, which are responsible for the deleterious effects of *B. anthracis* (12, 19–21). These anthrax toxins can facilitate the establishment of infection and lead to host mortality (13), contributing to early and late-stage infection. Thus, toxin neutralization can both prevent the establishment or stall disease progression, therefore, promoting host survival.

Development of specific antibodies to PA, LF and EF proteins have been demonstrated using an enzyme-linked immunosorbent assay (ELISA) following natural or experimental infection (14, 22–25). Toxin neutralizing antibodies also play an important role in conferring protection against anthrax in the host (14, 15). The toxin neutralization assay (TNA) is used to measure the capability of host serum to neutralize the cytotoxic effects of LT and ET on cells *in vitro* (14). The TNA quantifies only the functional subunit of the antibodies rather than the total anti-PA IgG antibodies detected by ELISA (14).

Antibody titres to *B. anthracis* diminish over time as reported in plains zebras (*Equus quagga*) that are naturally exposed, however it seems that frequent sublethal infections can boost antibody levels to maintain a detectable level of antibodies (26). The presence of neutralizing antibodies against anthrax lethal toxin has been reported in vaccine studies, with neutralizing antibodies positively correlated with anti-PA titres and increased survival rates (14, 22, 23, 27). Species differences in susceptibility to infection with anthrax have been reported (24). Some species like herbivores are highly susceptible, while carnivores and omnivores appear to be more resistant (25, 28). On the contrary, species that are resistant to spore challenge appear to be highly susceptible to intravenous toxin challenge and vice versa (7). However, no study has been conducted in free-living wild herbivores to see how toxin neutralization ability varies across species or between areas of higher or lower risk of anthrax exposure.

The *B. anthracis* lifecycle involves animal hosts, the external environment and potential mechanical vectors such as flies (29–32), vultures (e.g., *Gyps africanus*) (33–36), jackals (*Lupulella* spp.) and hyenas (*Crocuta crocuta*) (35, 37). Environmental factors influencing disease dynamics include soil properties such as calcium and pH, and weather factors such as rainfall, humidity, and temperature (7, 38–41). Anthrax is endemic to Kruger National Park (KNP) in South Africa and Etosha National Park (ENP) in Namibia. Southern Africa, including KNP and ENP, is considered the origin of anthrax (42). These two parks vary in anthrax incidence, with high and low incidence areas documented. Anthrax primarily affects grazing herbivores in ENP with plains zebra contributing to most of the mortalities (43), while in KNP, the primary host species over time has been greater kudu (*Tragelaphus strepsiceros*), a browsing herbivore. In ENP, browsers such as kudu account for about 1.7% of anthrax mortalities (44). In recent years in KNP, the seasonal timing and primary host species has shifted, to primarily wet season outbreaks affecting impala (*Aepyceros melampus*), a mixed grazing-browsing species (32).

The variation in anthrax ecology worldwide has served as an impediment for the blending of knowledge and outbreak forecasting (45) and therefore, identifying the variables that play a role in disease dynamics warrants substantial attention. Comparing two natural systems allows us to study the differences, patterns and pathways that may be unnoticed under the limited lens of a single system (46). In addition, comparing systems that differ in disease dynamics, but share the same potential host species, allows us to “control” for the large differences in ecology, behavior and immunity between different species, while exploring how exposure and immune response vary among populations of the same species. Before now, no research has been conducted to measure and compare the variability in *B. anthracis* exposure status or protection levels across different species and areas.

We investigated the variation in immune status among plains zebra and greater kudu in two different ecosystems (ENP, KNP) with different anthrax epidemiology. Specifically, we addressed the following questions: 1) Are serological patterns of host exposure to the anthrax bacterium concordant with spatial patterns of anthrax mortality from passive surveillance? 2) Does toxin neutralization ability vary based on species and/or environmental factors, such as frequency or dose of pathogen exposure? If this toxin neutralization is a species-level trait, then we would expect variation in the ability to tolerate or resist the effects of anthrax disease to be part of why species vary in their susceptibility to anthrax mortality, and that this ability would be consistent across study areas. However, if toxin neutralization varies based on pathogen exposure, then we expect to observe differences in neutralization ability for populations occurring in high or low anthrax incidence areas, where frequency of pathogen encounters by animals may vary. This study, therefore, investigated the immunological dynamics of anthrax infection in two national parks with a goal of understanding whether the rarity of disease mortality in an area is a function of low or no exposure or higher adaptive immune response. We examined the prevalence of exposure to the pathogen—as an index of exposure frequency—across host species and locations and evaluated how exposure relates to the ability of the host to mount an effective adaptive immune response, through the ability of hosts to neutralize the anthrax lethal toxin.

## Materials and Methods

### Study Areas

**Figure 1:**
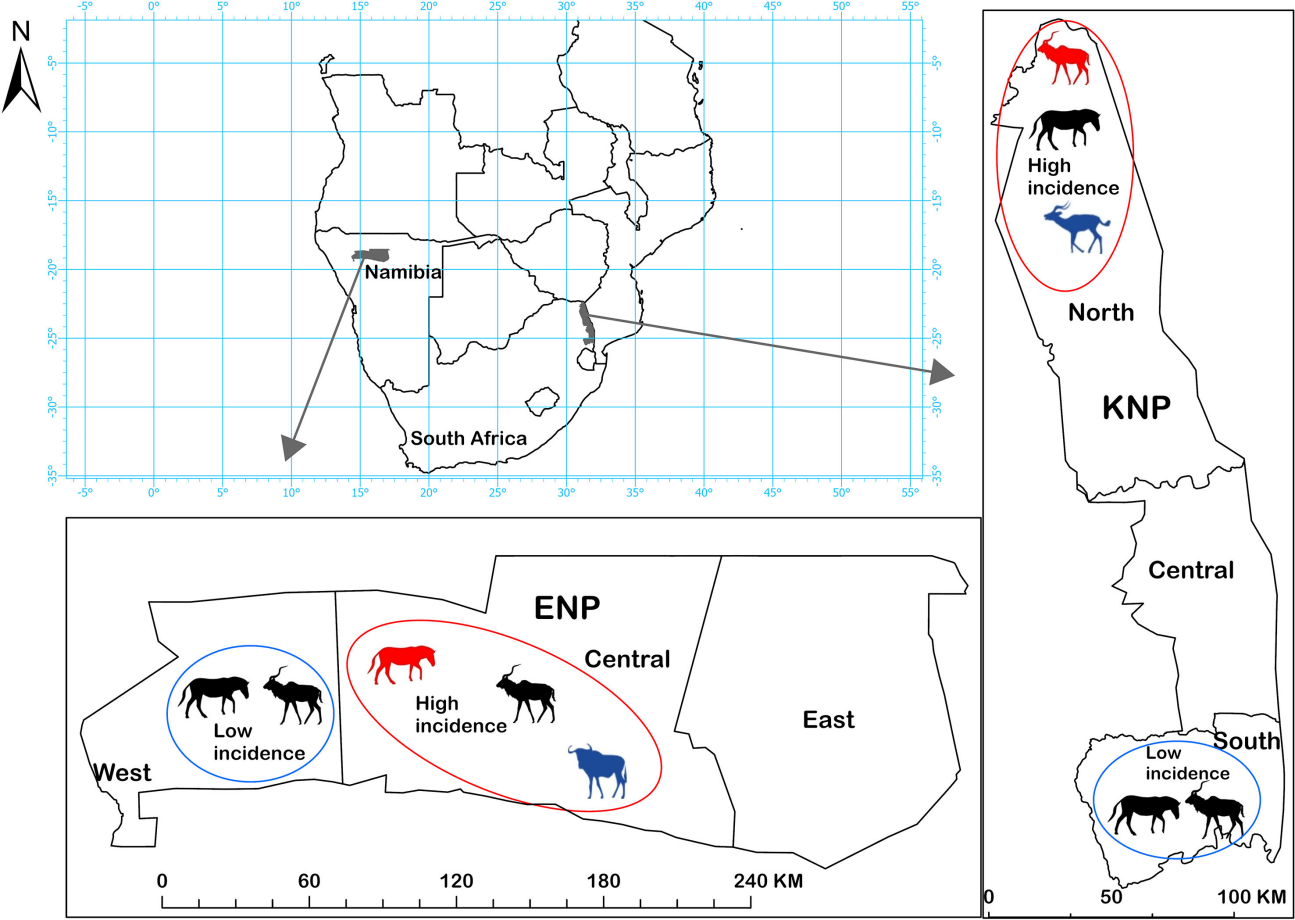
Etosha National Park (ENP) and Kruger National Park (KNP) in southern Africa, showing the study areas where anthrax outbreaks occur with high (red circles) or low (blue circles) incidence. Host species sampled for this study in different areas are shown with animal silhouettes. Kudu (*Tragelaphus strepsiceros*) and zebra (*Equus quagga*) were sampled in all four areas. Secondary host species were sampled in high incidence areas of each park: impala (*Aepyceros melampus*) in KNP, and wildebeest (*Connochaetes taurinus*) in ENP. The primary anthrax host species in a high incidence area is shown in red and the secondary host species in blue, otherwise, silhouettes are black. Assignment of areas as high or low incidence was based on anthrax mortality patterns observed in each park, and where anthrax occurs most commonly or least commonly, respectively.

This study compared serological evidence of *B. anthracis* exposure in host species in two large national parks. ENP (22,915 km^2^), Namibia, and KNP (19,485 km^2^), South Africa, are located nearly 2,000 km apart in southern Africa (**Figure 1**), a region considered the origin of anthrax (42). The anthrax endemic regions of these ecosystems are classified as arid savannas, based on annual rainfall less than 650 mm (47). Central ENP has an average rainfall of 358 mm (Okaukuejo weather station 1954-2020; 19.1669° S, 15.9171° E), is mostly an open shrubveld around a large salt pan. On the other hand, northern KNP is highly woody with grassland savannah (47), and an average rainfall of 430 mm. ENP is largely flat with some mountains in the far western part of the park while KNP has varying elevations, with Pafuri (found in the far northern part of KNP; 22.4206° S, 31.2296° E) having lower elevation flood-plains surrounded by higher elevations. In both parks, there are areas of high and low anthrax incidence (defined here as regular or infrequent anthrax occurrence over time, respectively). In KNP the high incidence area extends from Pafuri to Shingwedzi (23.1167° S, 31.4333° E) in the north and the low incidence area extends from Skukuza (24.9948° S, 31.5969° E) to Crocodile Bridge (25.3584° S, 31.8935° E) in the south. The high incidence area in ENP includes the central Okaukuejo management unit and the low incidence area include the western Otjovasandu (19.2300° S, 14.4800° E) management unit. These regions of low and high incidence were determined based on previous reports (35, 42) and the distribution of anthrax mortalities from historical data. Our study focused on plains zebra and greater kudu, sampled in high and low incidence areas of each park. For comparison, we included samples from a secondary anthrax host species in the high incidence area of each park: blue wildebeest (*Connochaetes taurinus*) in ENP and impala in KNP.

Anthrax primarily affects grazing and mixed-feeding herbivores. In the high incidence region of ENP (**Figure 1**), deaths of plains zebra and other herbivores climax at the closing of the rainy season, while African elephant (*Loxodonta africana*) deaths climax during the late dry season, though cases in all species can be observed sporadically throughout the year (35, 43, 48, 49). Seasonal outbreaks have been linked to differences in host foraging behavior altering exposure rates (26, 43, 44) and seasonal immune trade-offs (50). Zebra and wildebeest are grazing herbivores, kudu are browsing herbivores, and impala and elephant are mixed-feeding herbivores, which graze or browse depending on conditions. Plains zebras contributed to most of the mortalities in ENP followed by blue wildebeest (43). browsers, which include kudu, contributed the least (44).

In KNP, the main host species over time has been greater kudu, a browser contributing up to 75% of recorded cases from 1960-1990s (51). Anthrax was historically associated with dry seasons or droughts in KNP, occurring in explosive outbreaks on a roughly decadal cycle (29, 36, 51–53). Since 2008, smaller outbreaks have occurred annually and mainly in the wet season, and primarily affecting impala, a mixed grazing-browsing species (32). Exposure of browsing species has been hypothesized to occur *via* blowflies (*Chrysomya* spp.) feeding on anthrax carcasses, and then depositing *B. anthracis* spores onto the leaves of trees or shrubs near the carcass (54, 55). Plains zebra have contributed only 4% (44/962) of cases in KNP outbreaks (anthrax mortality reports from 1988-2016 obtained from the Skukuza Veterinary Services).

### Sample Collection

Serum samples were obtained from live animal captures from the four study species. We sampled 20 individuals per primary host species (zebra, kudu) per area, except for kudu in KNP (low incidence = 18, high incidence = 19). Twenty individuals per secondary host species were sampled only in high incidence areas of the parks where they occur (northern KNP: impala, n=20; central ENP: wildebeest, n=20). Negative and positive control serum samples were obtained by vaccinating two representative animals of each species (kudu, impala, zebra and wildebeest) in southern KNP. These animals were fitted with a satellite-GPS collar, sampled initially for the negative control, vaccinated with the Sterne live spore vaccine (Onderstepoort Biological Products, South Africa), and released. Each animal was vaccinated with 1 ml of Sterne spore vaccine intramuscularly as prescribed by the manufacturer. These animals were then recaptured after a month and serum samples were collected, which served as the positive controls. All ethical approvals were obtained from the University of Pretoria Research Ethics Committee, Animal Ethics Committee (REC 041-19) and the Department of Agriculture, Forestry and Fisheries (DAFF) in South Africa (Ref 12/1/1/18). Animals were immobilized following the “standard operating procedures (section 2.1.11) for the capture, transportation and maintenance in holding facilities of wildlife” by certified veterinarians and South African National Parks regulations and Namibian National Commission on Research, Science and Technology (authorization 2017070704) and the Ministry of Environment, Forestry and Tourism, Namibia. Also, approval was obtained from the University at Albany’s International Animal Care and Use Committee, approval numbers: 16-016, 18-013, 18-014, 18-015, 20-001.

### Mortality Data

Mortality data were analyzed to examine the distribution of *B. anthracis* positive cases and the distribution of mortality detection and reporting in each park (**Supplementary Table S1**). These data were collected as part of the opportunistic passive mortality surveillance in these parks. The data for KNP ranged from 1990-2015 and for ENP from 1996-2015, after restricting the time series to cases with GPS coordinates. These data, however, excluded a substantial number of kudu anthrax mortalities from when kudu dominated the outbreak cases. Carcasses were identified as anthrax positive following a positive result from blood smear examination, bacterial isolation or molecular detection (11, 32). Other information obtained included the date, locality, species and sex. For analysis, each park was grouped into three regions: for KNP, these are the northern, central and southern regions while for ENP, these are the western, central and eastern regions as defined by the park management (**Figure 1**). The mortality data were grouped into two causes of death: anthrax or others (e.g., predation, unknown). Anthrax important species for this analysis include zebra, impala, kudu and wildebeest (wildebeest was excluded from KNP and impala from ENP as they did not contribute significantly). All other species both for anthrax mortality and other causes of death were categorized as “others”; (for KNP other included mortalities from 57 different species, of which 21 species had anthrax mortalities, and for ENP included mortalities from 27 species, of which 6 species had anthrax mortalities). The mortality data were further used to confirm and distinguish between the high and low incidence areas of these parks.

### Anti-Protective Antigen (PA) Enzyme-Linked Immunosorbent Assay

In this study, serum samples were assessed for the presence of specific antibodies against the anthrax PA as described by Yu et al. (56), and Ndumnego et al. (23). Briefly, microtiter plates (Thermo Scientific™ Pierce 96-well Plates-Corner, USA) were coated overnight with 0.5 μg/ml rPA (List Biological Laboratories Inc., USA) in bicarbonate buffer at 4°C. Plates were washed twice with phosphate buffered saline (PBS) supplemented with 0.05% Tween-20 (Thermo Fisher Scientific, MA USA) (PBST) using a Biorad PW40 washer (Mamesla-Coquette, France). Plates were blocked with PBST supplemented with 5% skimmed milk powder (PBSTM) and then incubated for 1 h at room temperature. Plates were washed twice before the addition of duplicate test and control sera at a 1:40 dilution in PBSTM. This was followed by 30 min incubation on a rotatory incubator (Environmental Shaker-Incubator ES-20, Biosan Ltd, Germany). Afterwards, the plates were washed five times and recombinant protein A/G horseradish peroxidase (HRPO) conjugate (Pierce^®^ Protein A/G, USA) for zebra and wildebeest (57) and protein G HRPO conjugate (Invitrogen Protein G, USA) for impala and kudu were added to respective wells and incubated for 30 min on the rotary incubator. The binding of protein G HRPO to impala and kudu was evaluated in Supplementary methodology **Figure S1** and **Supplementary Table S2**. The plates were washed five times, after which the substrate 2,2’-Azinobis[3-ethylbenzothiazoline-6-sulfonic acid]-diammonium salt (ABTS) (Thermo Scientific USA) was added and incubated in the dark for 45 min. The absorbance was read at 405 nm using the Biotek Powerwave XS2 reader (USA). The ELISA results were interpreted as binominal data (positive/negative) with the threshold set at the mean plus three standard deviations (SD) of the negative control for the respective species. The optical density (OD) values were normalised per species to reduce variation between plates. Normalization between plates was done by calculating sample to positive (SP) ratios as the same positive control (for each species) was used on each of the plates. The binary outcome (positive/negative) was used to determine exposure while the SP ratios were used as a measure of the antibody response (23).

### Toxin Neutralization Assay (TNA)

The TNA was used to estimate the variation of anthrax LT neutralizing antibody among the different species in the two parks. The assay measures the ability of test sera to protect mouse macrophages from the cytotoxic effects of the toxin and is therefore not species-specific (14, 58).

The TNA was performed *in vitro* using J774A.1 mouse macrophage cell line (ECACC cat no 91051511), with modifications as described by Hering, et al. (27), and Ndumnego, et al. (59). Flat-bottomed 96-well culture plates (Corning ™, Corning incorporated, Germany) were seeded with 10^5^ mouse macrophage cells in 200 µL Dulbecco’s modified eagle media supplemented with 10% foetal bovine serum (TNA medium), and incubated at 37°C and 5% CO_2_ for 24 h. Duplicate test sera were diluted two-fold (1:50 to 1:6400) in TNA medium containing 500 ng/mL PA and 400 ng/mL LF (List Biological Laboratories Inc., USA). The sera and toxin were incubated for 1 h at 37°C and then transferred to the previously seeded cells and incubated for 3 h. Each plate also included 3 wells without cells as blanks, 3 wells for the toxin control and 2 wells for media control (used to calculate the neutralization titre). Each plate also contained a single dilution for the positive controls (to ensure consistency and reproducibility of the assay) for each animal species. Twenty-five µL of 3-(4,5-dimethylthiazol-2-yl)-2,5-diphenyltetrazolium bromide (Invitrogen, USA) was added to every well and incubated at 37°C and 5% CO_2_ for 2 h. The cells were lysed using a 100 µL mixture of 90% isopropyl alcohol, 0.5% sodium dodecyl sulphate (SDS), and 25 mM hydrochloric acid (HCl) followed by a 5 min incubation at room temperature.

The plates were read at an absorbance of 570 nm and the neutralization titres (NT) were calculated as:


$$NT=\frac{OD_{Sample}-OD_{Toxin\;control}}{OD_{Medium\;control}-OD_{Toxin\;control}}\times 100$$

The neutralization titre 50 (NT_50_) was calculated, using the Gen5 analysis software (Biotek Instruments, USA), as the highest titre that protected 50% of the macrophage cells. Samples that could not protect 50% of the cells were assigned an arbitrary value of 0.1.

### Statistical Analyses

Distributional patterns for total mortalities and anthrax mortalities were described for both parks to evaluate how serological results match with what is known about anthrax mortalities, based on disease surveillance. Anthrax mortalities for impala and wildebeest were only recorded in KNP and ENP, respectively. Mortality data from each park were plotted in ArcGIS pro version 2.8 and summarized as bar plots and maps.

We determined how host species differed in their immune responses (based on SP ratios) to *B. anthracis* between the two parks and between high incidence and low incidence areas using multivariable linear models coupled with the Tukey’s Honestly Significance Difference (HSD) test for multiple mean comparisons. Analyses were done separately for each species, and the SP ratios were log-transformed to normalize the data. The predictor variables included national park (KNP, ENP), area (high incidence or low incidence), LT neutralization status (positive, negative) and the interaction between national park and area. To compare exposure, we used logistic regression analysis with host exposure (positive or negative for anti-PA antibodies) as the response variable and park, area, and interaction between park and area as categorical independent variables.

To determine how the host species differed in their toxin neutralizing ability, a multivariable linear model with the Tukey’s HSD test for multiple mean comparisons was performed to evaluate whether national park (KNP, ENP), area (high or low incidence), host species (kudu, zebra), and level of anti-PA immune response (ELISA ODs), significantly predicted LT neutralization titres (NT_50_). Only TNA positive animals were included in the analysis, and NT_50_ and ELISA SP ratios were log-transformed first to normalize the data. To determine the difference in proportions of animals that neutralized the LT, logistic regression analysis was conducted to identify significant predictors for *B. anthracis* toxin neutralization ability (positive/negative status determined by TNA) in wild animal populations in ENP and KNP. Wildebeest and impala were not included in the regression analyses because these were sampled only from high incidence areas in ENP and KNP, respectively, but descriptive analyses for these species were performed.

The extent of agreement between the binary outcomes of anti-PA ELISA and TNA results separately for individual species (kudu=77, zebra=80, wildebeest=20 and impala=20) was determined using Spearman’s correlation and Cohen’s kappa (*k*) test (60). For this analysis, kappa ≠ 0, means that the agreement between anti-PA ELISA and TNA is different from chance agreement. The strength of agreement was assessed based on the criterion by Landis et al. (60), where <0 = poor; 0.01-0.20 = slight; 0.21-0.40 = fair; 0.41-0.60 = moderate; 0.61-0.80 = substantial; 0.81-1.00 = almost perfect.

All statistical analyses were done in R Console version 3.2.1 (61) with significance assessed at a 5% level.

## Results

### Mortality Distributions

**Figure 2:**
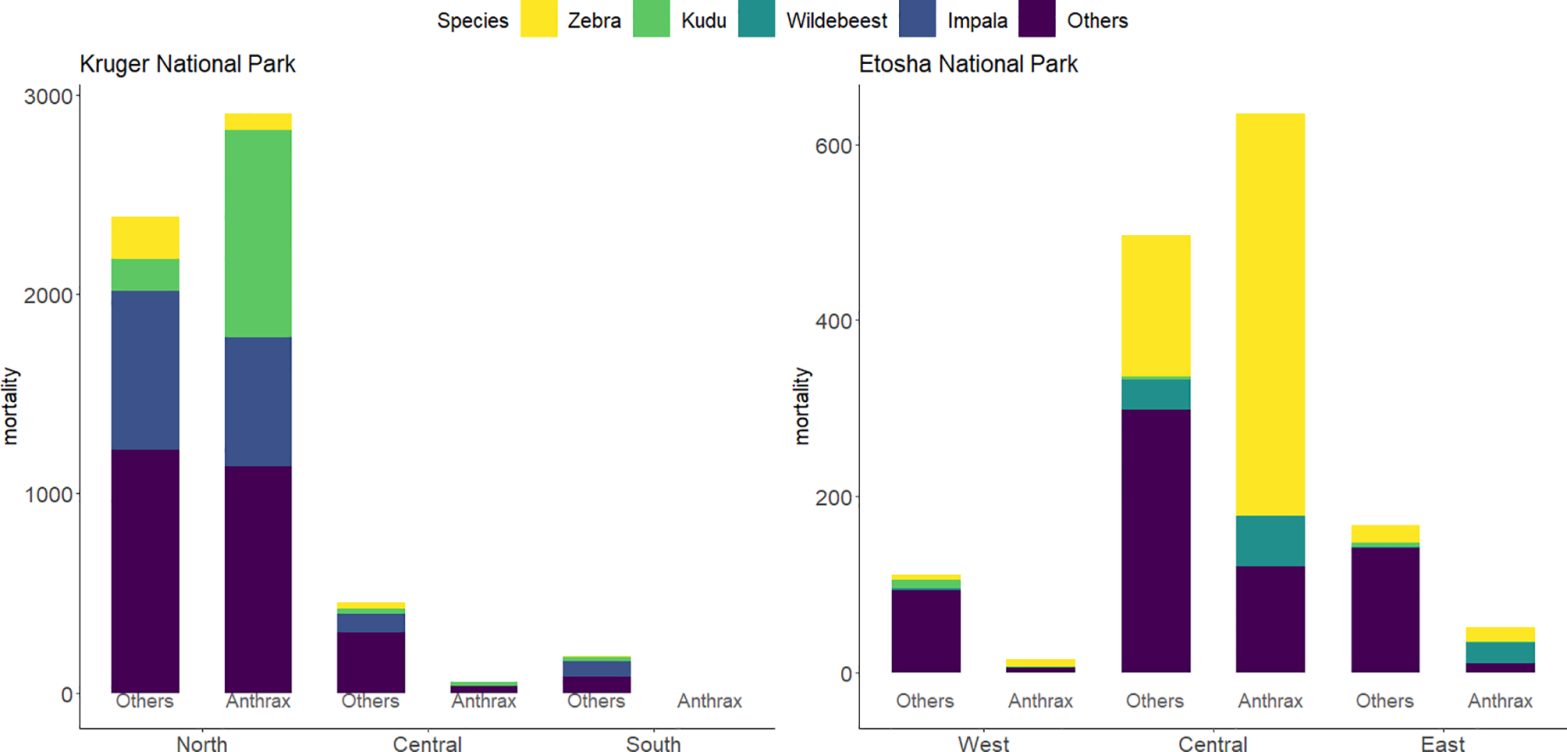
Bar charts of the distribution of mortalities by region and species from 1990-2016 in Kruger National Park (KNP) and from 1996-2016 in Etosha National Park (ENP). Mortalities are group into anthrax or other causes of death. Species of study included greater kudu (*Tragelaphus strepsiceros*), plains zebra (*Equus quagga*), impala (*Aepyceros melampus*), and blue wildebeest (*Connochaetes taurinus*). All other species included 21 species for anthrax mortalities and 57 species for non-anthrax mortalities (others) in KNP and 6 species for anthrax mortalities and 27 species for other mortalities in ENP. Data for KNP were provided by Skukuza Veterinary Services and for ENP from the Etosha Ecological Institute.

In ENP, the highest number of all mortalities (76%) were recorded in the central region, followed by 14.8% in the eastern region and 8.5% in the western region. In the central region, zebra contributed 54.6% (*N* = 618) of the total mortality, while wildebeest and kudu contributed 8.1% (*N* = 92) and 0.3% (*N* = 3), respectively (**Figure 2**). Zebra had the highest total mortality in the western and eastern regions (11.1% and 37%, respectively), followed by kudu in the west (7.9%) and wildebeest in the east (11.4%).

Of the anthrax mortalities observed in ENP, the highest number was recorded in the central region (90.4%), followed by 7.4% in the eastern and 2.1% in the western regions. Considering anthrax mortalities by species in ENP, the contribution to mortality for zebra was 68.7%, which was higher than wildebeest (11.7%), and kudu (0.3%). In the central region, zebra similarly contributed 72.0%, followed by wildebeest (9.0%), with no anthrax mortality recorded for kudu. Of the anthrax mortality in the east and the west, zebra contributed 32.7% and 53.3%, respectively, kudu 1.9% and 6.7%, respectively, and wildebeest contributed 46.2% in the east (**Figure 2**).

In KNP, the highest number of mortalities (88.4%) was recorded in the north, with most mortalities clustered around the Pafuri region, followed by 8.5% in the central region and 3.1% in the southern region. Mortalities in the northern region among the species of interest were dominated by impala (27.3%), followed by kudu (22.6%) and zebra (5.6%). For the central and southern part of KNP, impala contributed 18.8% and 43.2%, kudu, 7.5% and 10.8%, and zebra 6.3% and 2.7% respectively to the total mortality respectively (**Figure 2**). Of the total anthrax mortality in KNP, kudu contributed 35.0%, followed by impala (21.8%) and zebra (2.9%). Kudu made up 35.6% of the anthrax-related mortalities in the northern region, followed by impala (22.3%) and zebra (2.9%). In central KNP, impala contributed 1.9%, while kudu contributed 30.2% to the carcasses that were anthrax positive (**Figure 2**).

**Figure 3:**
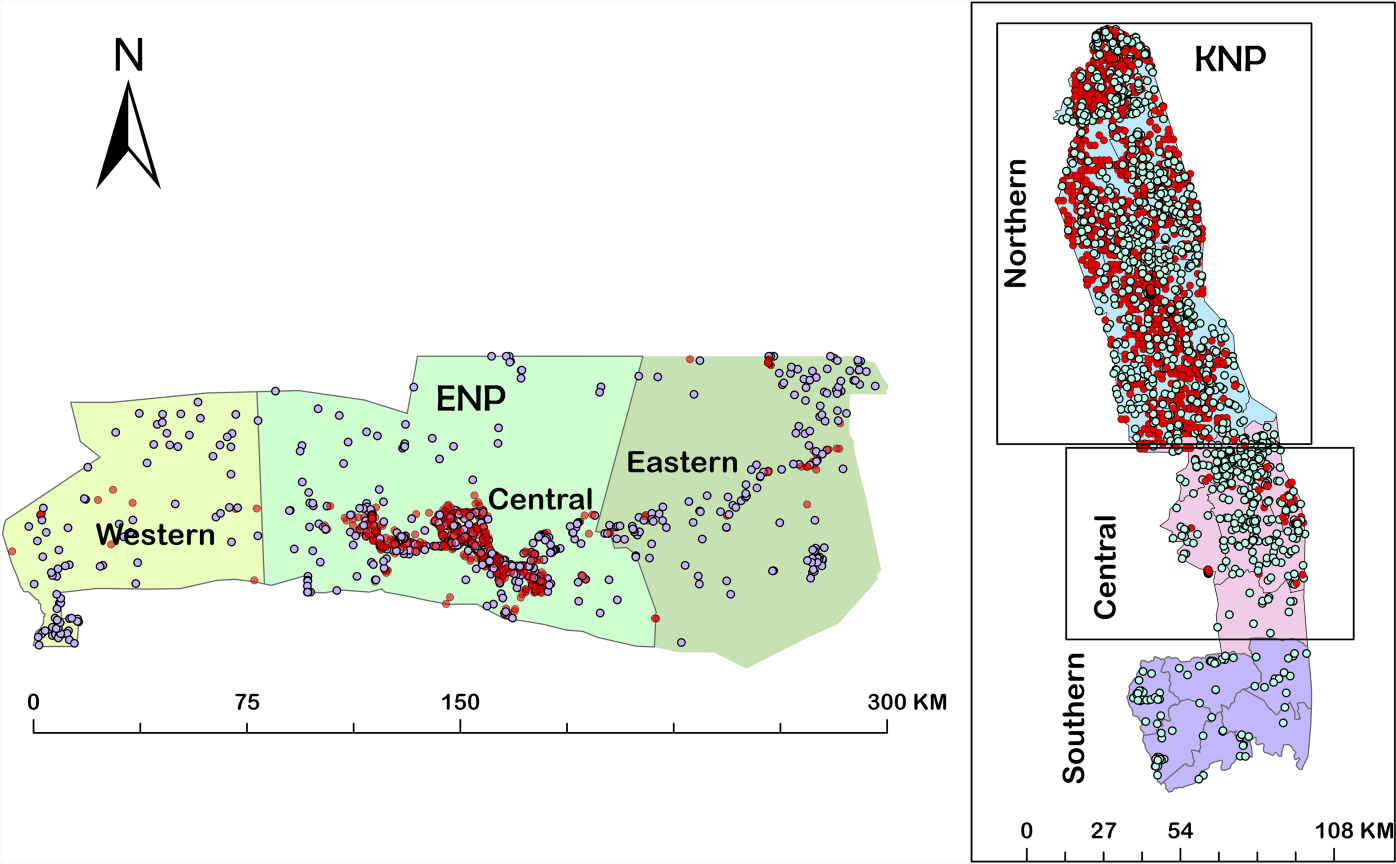
Maps showing distributions of mortalities from 1990-2015 in the three regions of Kruger National Park (KNP), South Africa and from 1996-2016 in the three regions of Etosha National Park (ENP), Namibia. Red dots indicate anthrax positive mortalities and the white dots indicate non-anthrax mortalities.

These patterns confirm our expectations that zebra in ENP and kudu in KNP are the primary host species in these systems, and that they are minor hosts in the other park (i.e., zebra in KNP and kudu in ENP). The distribution of anthrax mortalities revealed that the central part of ENP (90.5%) and northern part of KNP (98.2%) are the most affected over the years, followed by the eastern part of ENP (7.4%) and the, western part of ENP (2.1%) and central region (1.8%) in KNP, with no positive cases observed in the southern region of KNP (**Figure 3**).

### Exposure to *B. anthracis*

**Figure 4:**
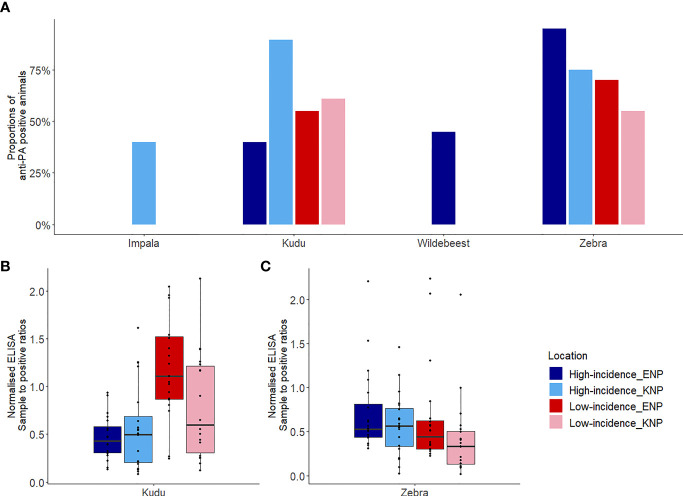
Host exposures to *Bacillus anthracis* assessed based on anti-protective antigen (PA) antibody titres. **(A)** The proportion of each host species that was seropositive for anti-PA antibodies, as determined using enzyme-linked immunosorbent assay (ELISA), by area. **(B, C)** Box plots showing sample to positive (SP) ratios for antibodies against PA for **(B)** kudu (*Tragelaphus strepsiceros*) in each park and area, and **(C)** zebra (*Equus quagga*) in each park and area. Kudu and zebra were sampled from high incidence and low incidence areas of Kruger National Park (KNP) in South Africa and Etosha National Park (ENP) in Namibia, while impala (*Aepyceros melampus*), and wildebeest (*Connochaetes taurinus*) were sampled from only the high incidence area of KNP and ENP, respectively. Box plots **(B, C)** were separated to avoid comparison between species as the technique utilized is species-specific. The locations of high and low incidence areas in each park are shown in **Figure 1**.

Host species showed significant differences in exposure to *B. anthracis* between parks, based on anti-PA antibody response. Kudus in KNP had significantly higher exposure to *B. anthracis* than kudus in ENP (*p* = 0.005, **Table 1** and **Figure 4A**). The kudus in KNP had significantly higher odds of exposure to *B*. *anthracis* than those in ENP (odds ratios (OR) = 2.9, *p* = 0.005). Zebra in ENP also had higher odds of exposure to *B*. *anthracis*, with a higher proportion of anti-PA ELISA positives, than those in KNP (OR = 3.1, *p* = 0.005, **Table 1** and **Figure 4C**). Details for all four species are shown in **Table 1** and **Figure 4A**.

**Table 1:** Differences in host exposure to *Bacillus anthracis* by species and location, assessed through anti-protective antigen (PA) antibodies.

Animal species	National park	Location	No. of animals	% of positive animals (N)	Mean SP ± SD for positive animals	Odds ratio of exposure	*p*-value
Kudu	ENP	High incidence	20	70 (14)	0.59 ± 0.29		0.94^a^
							0.51^b^
		Low incidence	20	60 (12)	0.71 ± 0.58		
		Whole park	40	65 (26)	0.65 ± 0.46		
	KNP	High incidence	19	94.7 (18)	1.54 ± 0.29		0.04^a^
							0.09^b^
		Low incidence	18	72.2 (13)	1.02 ± 0.39		
		Whole park	37	83.8 (31)	1.24 ± 0.74	2.9^c^	0.005^a^
							0.06^b^
Zebra	ENP	High incidence	20	95 (19)	0.73 ± 0.48		0.97^a^
		Low incidence	20	70 (14)	0.66 ± 0.58		0.10^b^
		Whole park	40	82.5 (33)	0.69 ± 0.53	3.1^c^	0.07s
							0.04^b^
	KNP	High incidence	20	75(15)	0.66 ± 0.33		0.03^a^
							0.09^b^
		Low incidence	20	50 (10)	0.41 ± 0.46		
		Whole park	40	62.5 (25)	0.53 ± 0.40		
Wildebeest	ENP	High incidence	20	35 (7)	0.52 ± 0.23		
Impala	KNP	High incidence	20		0.48 ± 0.19		

Optical density (OD) values were measured using an anti-PA ELISA, and mean sample to positive (SP) ratios were estimated for all sampled animals in a given location. SD is the standard deviation. Areas of high and low incidence in each park (ENP, Etosha National Park and KNP, Kruger National Park) are shown in **Figure 1**. The species of study included greater kudu (Tragelaphus strepsiceros), plains zebra (Equus quagga), impala (Aepyceros melampus), and blue wildebeest (Connochaetes taurinus).

a p-value for comparison of mean anti-PA OD.

b p-value for comparison of proportion of positive animals.

c Odds ratio comparing national parks for each species.

Host species had higher antibody response (based on SP values) in the park where they were considered the primary anthrax host, but lower response where they were the secondary anthrax host. Kudus in KNP had significantly higher (1.3 times, *p* = 0.047) anti-PA ELISA response (1.24 ± 0.74) than those in ENP (0.65 ± 0.46), but exposure in the high versus low incidence areas (irrespective of park) was not statistically different (*p* = 0.41, **Supplementary Table S3**). Zebras in ENP had significantly higher (1.3 times, *p* = 0.034) anti-PA ELISA response (0.69 ± 0.53) than those in KNP (0.53 ± 0.40), while differences between the high versus low incidence areas (irrespective of park, *p* = 0.29) were statistically insignificant. The interaction between national park and area contributed significantly (*p* = 0.015) to the level of immune response in this study. When SPs were compared between incidence areas within parks separately for each species, there was a significant difference for kudu (**Table 1** and **Figure 4B**) and zebra (**Table 1** and **Figure 4C**) in KNP, but not in ENP. In the high incidence areas, the average anti-PA SPs for KNP impala and ENP wildebeest were 0.52 ± 0.23 and 0.48 ± 0.19, respectively (**Table 1**).

### Neutralization of Anthrax Lethal Toxin

**Figure 5:**
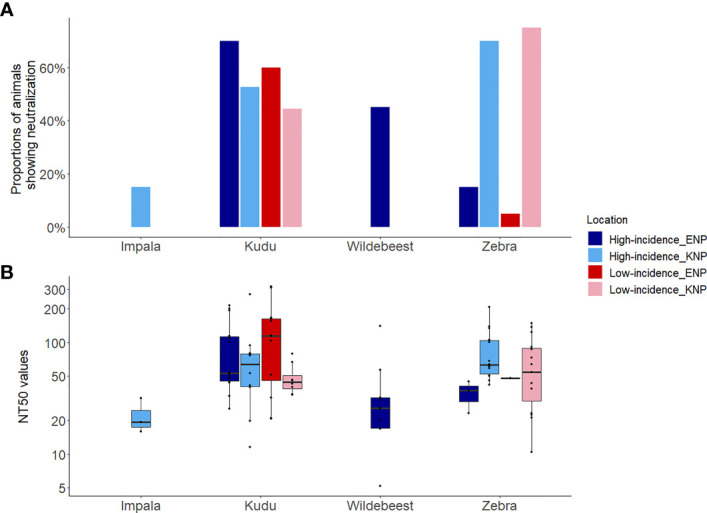
Host toxin neutralization against the *Bacillus anthracis* lethal toxin for four wild herbivore species sampled in Kruger National Park (KNP), South Africa, and Etosha National Park (ENP), Namibia, showing **(A)** the proportion of animals showing neutralization, and **(B)** the neutralization titre 50 (NT_50_). The NT_50_ was the highest titre that protected 50% of mouse macrophage cells. The y-axis of plot **(B)** represents log10 transformed NT_50_. Species of study included greater kudu (*Tragelaphus strepsiceros*), plains zebra (*Equus quagga*), impala (*Aepyceros melampus*), and blue wildebeest (*Connochaetes taurinus*). The locations of high and low incidence areas in each park are shown in **Figure 1**.

The distribution of hosts (kudu and zebra) by park had a significant influence on the serum-LT neutralization titres (**Supplementary Table S4**). Kudus in both parks and zebras in KNP all showed significantly higher odds of *B*. *anthracis* toxin neutralization (> 45%) than zebras in ENP (10%, 4/40; *p* = 0.001) (**Figure 5**). The ability to neutralize the toxin pooled for all species across parks did not significantly differ by area (high incidence area = 51.9% (41/79); low incidence = 46.2% (36/78), *p* = 0.47). Further analyses of the association of toxin neutralization proportion and park (irrespective of incidence status) showed a significantly higher proportion of zebra neutralizing the anthrax LT in KNP than in ENP (*p* = 0.001; **Figure 5A**). In contrast, a higher proportion of kudu in ENP was able to neutralize the anthrax toxin than kudu in KNP, although the difference was not statistically significant (*p* = 0.15; **Figure 5**). Only 3/20 impala showed toxin neutralization, while 9/20 wildebeest neutralized the toxin (**Figure 5A** and **Table 2**).

**Table 2:** Lethal toxin (LT) neutralization titres and proportion of herbivores that neutralized anthrax LT in Kruger National Park (KNP), South Africa, and Etosha National Park (ENP), Namibia.

Animal species	National park	Location	No. of animals sampled	% of positive animals (N)	Mean NT_50_ ± SD of TNA positive animals	Odds ratio	*p*-value
Kudu	ENP	High incidence	20	70 (14)	92.0 ± 66.7		0.56^a^
		Low incidence	20	60 (12)	130.9 ± 100.5		0.95^b^
		Whole park	40	65 (26)	110.0 ± 84.6	1.96^c^	0.03^a^
							0.02^b^
	KNP	High incidence	19	52.6 (10)	100.9 ± 73.5		0.61^a^
							0.06^b^
		Low incidence	18	44.4 (48)	59.6 ± 16.1		
		Whole park	37	48.6 (18)	64.2 ± 56.3		
Zebra	ENP	High incidence	20	15 (3)	35.2 ± 10.9		0.29^a^
							NA^b^
		Low incidence	20	5 (15)	47.8 ± NA		
		Whole park	40	10 (4)	38.3 ± 10.9		
	KNP	High incidence	20	70 (14)	85.0 ± 47.6		0.45^b^
		Low incidence	20	75 (15)	66.3 ± 43.9		0.72^a^
		Whole park	40	72.9 (29)	75.3 ± 45.9	23.7^c^	0.05^a^
							0.14^b^
Wildebeest	ENP	High incidence	20	40 (8)	38.5 ± 40.9	NA	NA
Impala	KNP	High incidence	20	15 (3)	22.3 ± 8.3	NA	NA

The location of high and low incidence areas in each park are shown in **Figure 1**. TNA is the toxin neutralization assay; SD is the standard deviation, and the species of study included greater kudu (Tragelaphus strepsiceros), plains zebra (Equus quagga), impala (Aepyceros melampus), and blue wildebeest (Connochaetes taurinus). The neutralization titre 50 (NT_50_) was the highest titre that protected 50% of mouse macrophage cells.

a p-value for comparison of mean of neutralization titre 50 (NT_50_).

b p-value for comparison of the proportion of animals that showed neutralization.

c Odds ratio comparing national parks for each species.

NA stands for not applicable.

When considering only TNA-positive animals (animals that showed neutralization), kudus in ENP also had significantly higher titres (110.0 ± 84.6) than those in KNP (64.2± 56.3; *p* = 0.03, **Table 2** and **Figure 5B**). For zebra, NT_50_ were higher in KNP (75.3 ± 45.9) than ENP (38.3± 10.947) (regardless of area, *p* = 0.05). The titres of the two host species were also compared within the national parks, pooling across low and high incidence areas, which revealed that there was not a statistically significant difference in neutralizing titres between kudu and zebra in KNP (64.2 vs. 75.3; *p* = 0.072); in contrast, titres significantly differed between the two species in ENP (110.0 vs. 38.3, respectively; *p* = 0.03) (**Table 2** and **Figure 5B**). NT_50_ levels for impala and wildebeest were lower than zebra and kudu in all locations (**Table 2**).

### Relationship Between Pathogen Exposure and Toxin Neutralization

Kudu showed a statistically significant and moderate agreement between anti-PA and TNA (kappa = 0.47, 95% CI: 0.28-0.66, *p* = 0.0001). There was a slight agreement between these measures for zebra (kappa = 0.096, 95% CI: 0.089-0.19), but this was not significant (*p* = 0.213). For wildebeest there was a fair agreement (kappa = 0.381, 95% CI: -0.02-0.78, *p* = 0.081) and for impala no agreement (kappa = -0.195, 95% CI: -0.42-0.036, *p* = 0.253), but neither species showed statistical significance (**Table 3**).

**Table 3:** Comparison of anti-protective antigen (PA) enzyme-liniked immunosorbent assay (ELISA) and toxin neutralization assay (TNA) for the detection of immune exposure to *B. anthracis* in kudu, zebra, wildebeest and impala from Kruger (KNP) and Etosha (ENP) National Parks in South Africa and Namibia, respectively.

Species	National Park	Sample number	ELISA Status	TNA		*p*-value
				No. negative (%)	No. positive (%)	
Kudu	ENP	40	Negative	12 (30.0)	9 (22.5)	0.002
			Positive	2 (5.0)	17 (42.5)	
	KNP	37	Negative	8 (21.6)	1 (2.7)	
			Positive	11 (29.7)	17 (45.9)	0.012
	Total	77	Negative	20 (26.0)	10 (13.0)	0.001
			Positive	13 (16.9)	34 (44.1)	
Zebra	ENP	40	Negative	7 (17.7)	0	
			Positive	29 (72.5)	4 (10.0)	0.437
	KNP		Negative	9 (22.5)	5 (12.5)	
			Positive	2 (5.0)	24 (60.0)	<0.001
	Total	80	Negative	16 (20.0)	5 (6.3)	
			Positive	31 (38.7)	28 (35.0)	0.049
Wildebeest	ENP	20	Negative	9 (45.5)	2 (10.0)	
			Positive	2 (10.0)	7 (35.0)	0.022
Impala	KNP	20	Negative	10 (50.0)	2 (10.0)	
			Positive	7 (35.0)	1 (5.0)	0.656

The species of study included greater kudu (Tragelaphus strepsiceros), plains zebra (Equus quagga), impala (Aepyceros melampus), and blue wildebeest (Connochaetes taurinus).

**Figure 6:**
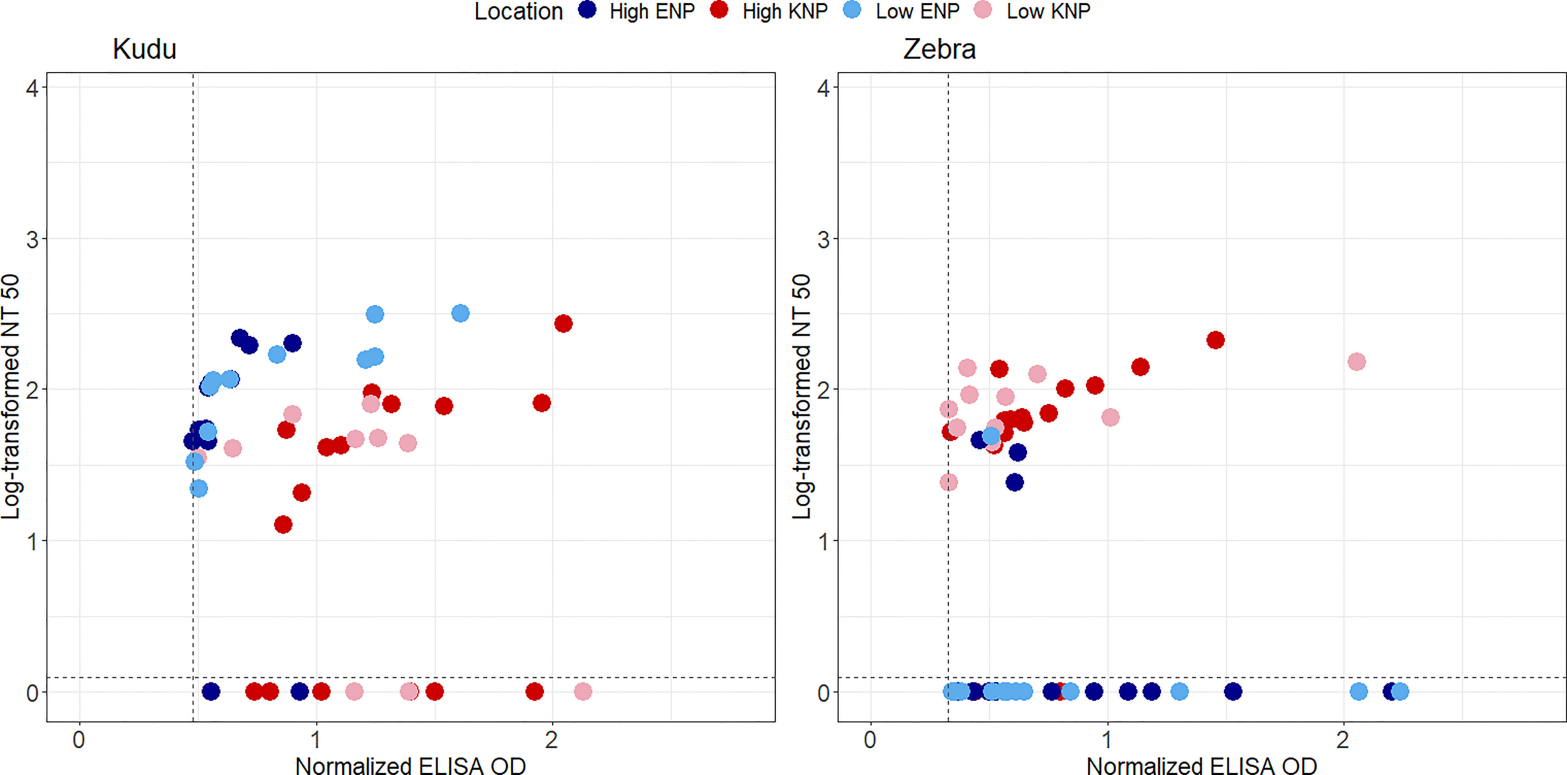
Scatter plots representing log-transformed neutralization titre 50 (NT_50_) and normalised anti-protective antigen (PA) enzyme-linked immunosorbent assay (ELISA) optical densities (ODs) for greater kudu (*Tragelaphus strepsiceros*) and plains zebra (*Equus quagga*) from Kruger National Park (KNP) in South Africa and Etosha National Park (ENP) in Namibia. The NT_50_ was calculated as the highest titre that protected 50% of the mouse macrophage cells. Shapes and colors of marker points represent different parks (blue circle: ENP, red triangle: KNP), and variation in color shades indicate study area that differs by anthrax incidence (dark: high incidence, light: low incidence) in each park. TNA negative sera (samples that could not protect 50% of the macrophages) are seen below the dotted horizontal lines in each plot. Only samples that were anti-PA positive are shown as others were assumed to not have been exposed to *Bacillus anthracis* (the threshold for anti-PA positive is shown with the dotted vertical line). The locations of high and low incidence areas in each park are shown in **Figure 1**.

There was a medium and significant positive correlation between anti-PA titres and TNA values, using Spearman’s correlation (rho = 0.40, *p* = 0.001). A correlation in kudu in both parks and zebra in KNP (**Figure 6**) provided evidence for saturation in TNA values as SP values increased.

## Discussion

In this study, we examined anthrax PA-specific and anthrax LT neutralizing antibodies to compare immune exposure and response to *B. anthracis* in four wildlife species. This study reveals a wide presence of anti-PA antibodies in the various host species sampled. It was seen that the spatial patterns of anthrax mortality from passive surveillance from both parks reflect the serological patterns of exposure to *B. anthracis*. Interestingly, even though these parks share similar host species, there were significant differences in the proportions of animals that tested positive for anti-PA antibodies and level of antibody response (SP) between the two parks. Also, we noted that toxin neutralizing ability is not necessarily a trait of species, but a product of environmental factors and exposure, which include access to the pathogen, frequency of exposure, and/or the dose of exposure. This study also represents the first report of neutralizing titres in wild herbivores.

### Spatial Patterns in *B. anthracis* Exposure and Anthrax Mortality

Spatial patterns in *B. anthracis* exposure agreed with the anthrax mortality patterns in both parks. The mortality data from each park showed zebra and wildebeest in ENP and kudu and impala in KNP as the most affected species in their respective parks. This is in accordance with previous reports from both parks that show that these species have the highest anthrax mortality in these parks (43, 44, 51). Most of the anthrax mortalities in KNP were from the northern part of the park, agreeing with the high incidence status previously attributed to this region of the park (36). Fewer mortalities were found in the central region, but no positive anthrax cases were found in the southern part of KNP. This result strengthens the divide between the high incidence and low incidence areas of KNP. However, we found that >50% PA positive animals were reported in the low incidence area. The absence of anthrax mortality in the southern part of KNP could result from sampling bias, as a relatively low proportion of overall mortalities were from this part of the park. Also, it has been reported that relying on carcass discovery or passive surveillance might not give the true picture of exposure in a population (62). For ENP, most of the anthrax mortalities were found in the central part of the park, with very few cases found in the east and the west. Unlike in KNP, the western (low incidence) part of ENP had some anthrax cases, which suggests possible exposure in this part of the park as supported by the moderately high prevalence of anti-PA antibodies found in both zebra and kudu (50-60%) in this region.

The anti-PA antibodies reported in this study indicated that the animals in these parks are exposed to varying doses of *B. anthracis* spores and/or repeated exposures in the environment and can mount an effective adaptive immune response. These results build on existing evidence that herbivores exposed to sublethal doses of *B. anthracis* in the environment develop antibodies against the pathogen (26). Moreover, this claim contradicts previous studies suggesting herbivores in anthrax high incidence regions are susceptible and naïve to *B. anthracis* and die following severe and sudden exposure. These assumptions of previous studies were based on a lack of detectable anti-PA titres (25, 28). However, the current study and previous studies each used a different serological method, namely indirect anti-PA ELISA (this study), QuickELISA kit (Anthrax-PA kit, Immunetics, Incorporated, USA) (25) and competitive indirect anti-PA ELISA (28), which could account for the different results. The competitive indirect anti-PA ELISA, unlike the indirect anti-PA ELISA, requires a high quantity of antibodies for there to be a 0.2 OD difference between two consecutive dilutions due to the inhibited counterpart and is thus less sensitive than the latter (26). The Quick ELISA kit also lacks the sensitivity to detect animals with low antibody titres (26). The indirect ELISA used in our study is not without its limitations. The conjugate will only optimally bind for specific species for which they were developed and for closely related species (57, 63). In this study, protein A/G conjugate was used for zebra and wildebeest while protein G was used for kudu and impala, which were selected based on a preliminary study (**Supplementary Figure S1**). These differences in binding specificities make it unsuitable to compare antibody titres between species, but comparisons between locations within a species remain robust. There are varying reports of the binding ability of the commercially available conjugates in these wildlife species (63, 64) and therefore species-specific conjugates to overcome this limitation are needed.

Sublethal exposure, and how frequently hosts encounter the pathogen, may have impacts on host immunity and disease dynamics (24, 26, 65). Kudu in the two parks showed a relatively high prevalence of pathogen exposure (65% in ENP and 84% in KNP), yet unlike KNP, kudu anthrax mortality in ENP is rarely observed. Thus, kudu in ENP may be commonly exposed to the pathogen, but in lower doses unlikely to cause mortality. Also, it has been shown in a previous study that an animal host may ingest a high number of spores that pass through the digestive tract without any invasion or that may cause a sublethal infection (66). Our study reported kudu in KNP are significantly more likely to be exposed to the pathogen than their counterparts in ENP and make up about 75% of historical anthrax cases (51) and 35.6% of the recorded cases from 1990 in KNP. In contrast, kudu in ENP contribute only 0.3% of recorded cases (**Figure 2**) in this study and this was reflected in the anti-PA antibody prevalence.

Both kudu and zebra in the two parks had antibodies against *B. anthracis* PA, though differences in antibody prevalence corroborate species and regional differences in anthrax incidence. Anthrax outbreaks in kudu in KNP have been linked to dissemination by blowflies in the park (32, 67). Hugh-Jones, et al. (36), indicated that *Chrysomya* spp. blowflies feeding on anthrax carcasses in KNP deposit *B. anthracis* spores onto the leaves of trees or shrubs near the carcass at the height that kudu feed, thereby creating a higher inoculum and exposure for the kudu in KNP. The increase in *B. anthracis* inoculum by the *Chrysomy*a flies on shrubs eaten by browsers in KNP might cause the higher mortality rates reported for browsers in KNP compared to ENP. A blowfly transmission pathway has not been detected in ENP. While Nalisa (68) recorded the presence of *B. anthracis* in flies of the *Muscidae* and *Calliphoridae* families, these flies were observed in relative low density at carcass sites in ENP. However, because of high vertebrate scavenger activity in ENP, most carcasses are consumed before flies can reproduce (37). This suggests that kudus in ENP can be exposed to the pathogen, but possibly to a lesser extent due to a smaller amount of dissemination and bacterial inoculum, through other mechanical vectors depositing the spores onto the leaves of trees or shrubs (31, 55, 68).

In ENP, anthrax affects mainly grazers rather than browsers (43). Although this is supported by the low levels of anthrax mortality in ENP browsers, the anti-PA antibodies indicate that kudu in ENP are exposed to the *B. anthracis* spores in the environment and this may require further investigation. Furthermore, the low number of kudu cases reported in ENP over the years (44, 69) might be underreported as the species occurs primarily in inaccessible woodlands, which exist outside of the central open plains region (70, 71), leading to reduced mortality surveillance in these habitats. Despite these differences in surveillance effort, Huang et al. (72), reported that open habitats in ENP have higher anthrax risk than the woodland habitats preferred by kudu.

Zebra in ENP had significantly higher antibody responses, as indicated by the anti-PA ELISA, than zebra in KNP (**Figure 4C** and **Table 1**). The high proportion of zebra (82.5%) in ENP testing positive for anti-PA antibodies in this study was similar to Cizauskas, et al. (26), who demonstrated a 52-87% prevalence of anti-PA antibodies in ENP zebra. This prevalence is reflected by zebra making up 68.7% of the anthrax mortalities in ENP compared to only 2.9% in KNP (**Figures 2**, **3**). In previous studies conducted in ENP and Serengeti National Park, Tanzania, none of the zebras tested positive (25, 28). The difference between the exposures and antibody levels in the two populations of zebras could be associated with the spore concentration in the soil ingested during grazing (43, 73), or interactions between zebra diet and foraging behavior, which may alter exposure risk over time (44).

Based on our results, kudu in KNP and zebra in ENP encounter lethal doses of the pathogen in the environment more often than other species in these parks, resulting in the higher mortality rates, seen in the mortality reports. These exposure differences may arise from behavioral and ecological factors as well as climate extremes such as droughts and flooding (74). Furthermore, the season of anthrax outbreaks between the two parks (43, 51) may contribute towards the difference observed between animal species in the two parks. The mortality and exposure results confirm that kudu in KNP and zebra in ENP are the most affected species in each park, followed by impala for KNP and wildebeest in ENP (32, 43, 51) (**Figure 4A** and **Table 1**).

The animals in the high incidence region of KNP had higher antibodies titres as reflected by their anti-PA antibody response than animals in the low incidence region of the park. These animals are 2.8 times more likely to be seropositive for *B. anthracis* anti-PA antibodies than animals in the low incidence region of the park. The presence of physical barriers such as rivers restrict the long-range movement of animals (personal communication, Skukuza State Veterinary Services, O. Louis van Schalkwyk) and may explain the difference in exposure. Also, home range sizes may be much smaller in KNP (Huang, unpublished data). We speculate that animal movement may restrict spore distribution and therefore may be responsible for the difference noted. Also, differences in animal densities and wild ungulate community composition could influence the variation seen in this study, and this requires further study. The finding of seropositive zebras and kudu in southern KNP indicates that animals are also exposed in the “low-incidence” area. Steenkamp et al. (75), identified the “low-incidence” area in KNP as a region of high *B. anthracis* spore suitability. Also, previous anthrax reports from KNP show that large anthrax outbreaks in the 1960s spread from the northern area south to the central part of KNP (53). There was an obvious bias in the passive surveillance of KNP as seen in the disparity between samples submitted from the north and south (**Figure 2**). Also, a similar bias was noticed in ENP where mortalities, in general, were underreported in both the western and eastern regions of the park (**Figure 2**).

In ENP there was no significant difference in anti-PA antibodies in animals in the high and low incidence regions. The absence of spatial patterns in exposure could be because ENP does not have physical barriers (such as rivers) that would prevent or slow movement between the west and central regions of the park, and thus animals can move across regions (76, 77). Secondly, animals in ENP have relatively large home ranges, and animals sometimes move between the western and central parts of the park (Huang, unpublished data). A study suggested that spores could concentrate more in the waterholes dispersed in the western part of ENP, as 26% of waterholes in the western part tested positive for anthrax spores (35), although Turner et al. (78), found that spore concentrations in waterhole sediments are too low for lethal exposures. Cloete (79) reported that there was no significant difference in spore survival by soil types sampled from different regions of the park. Together, these results suggest that the whole park may be a suitable habitat for *B. anthracis* especially when there are no physical barriers (beyond the salt pan) to restrict herbivore movement or spore distribution. Thus, most of ENP could potentially be high incidence for anthrax, but cases in the west may be underreported due to lower surveillance effort over time. Surveillance could be more evenly applied in both parks, to examine whether the serological patterns observed here are evidence of unreported anthrax cases/outbreaks or sublethal exposures to spores that do not lead to mortalities.

Based on results of this study, different herbivore species in the same ecosystem could be affected at different times and different rates, based on differences in their ecology or behavior. Outbreaks in zebra populations have been shown to occur mostly during the wet season or towards the end of the rainy season, with some cases occurring during droughts or extended dry periods (80, 81). In contrast, outbreaks in kudu occur largely during the dry season as seen in KNP and other parks (29, 51–53, 82). The grazing versus browsing transmission pathways occur at different timescales, which may have important effects on disease dynamics, pathogen diversity, and host resistance. Browsing-based transmission should occur shortly after host death before rainfall or leaf loss by deciduous trees/shrubs reduces exposure (69). Grazing-based transmission occurs only upon the regeneration of vegetation at a carcass site, and continues for years, with exposure dose decaying over time (73, 78).

### Species and Spatial Patterns in Toxin Neutralization Ability

Spatial patterns in toxin neutralization suggest that environment (affecting exposure frequency or dose) and the presence of neutralizing antibodies are the major determinants of the animal’s tolerance to the LT. Kudu and zebra demonstrated interesting variation in levels of neutralization. Kudu in ENP had a higher TNA response than kudu in KNP. Similarly, zebra in KNP had a higher TNA response than zebras in ENP. These results agree with the mortality records of these species in the two parks (43, 44, 51). Based on mortality patterns and exposure prevalence, we can assume that zebra in ENP and kudu in KNP are exposed more often, and to larger doses, than in the other park. Thus, those host populations with lower mortality (kudu in ENP and zebra in KNP) are more likely to be exposed to sublethal amounts of the pathogen based on their foraging behavior and the relative risk of that behavior in the two landscapes (43, 44, 51), yet show greater toxin neutralization than their counterparts in the other park. A previous study showed that animals that were immunized with antigens of spore origin conferred protection against *B. anthracis* through the production of antibodies that reduced spore germination (83). This type of sublethal natural “immunization” may have induced anti-spore antibodies and reduced germination in zebra in KNP and kudu in ENP (84), but this hypothesis would need further investigation.

The production of high-affinity memory B-cells during affinity maturation in the germinal centres is very important in the stimulation of an effective immune response (85, 86). When the concentration of the antigen is high or encountered more frequently, this leads to low competition among B-cells and the germinal centres become occupied with producing antibodies that have a lower affinity (85–87). Dumas et al. (88), also suggested that a higher immune response is derived from severe disease caused by exposure to a high amount of antigen over longer periods. Zebra in KNP and kudu in ENP could be better protected from the effect of the LT (89, 90), which may be due to their ability to develop antibodies of high affinity (14). As discussed earlier, a relationship has been established between antigen dose, “immunization” (exposure) interval and development of antibodies with high affinity (85, 87). This relationship may play a role in animals with higher neutralization that may have moderate doses and at longer intervals. It is important to note that no study has been conducted on affinity maturation with relation to dose in natural systems. Verma et al. (91), suggested that characteristics of the antibodies (factors such as the species of origin, subclasses and isotype) being examined in the test could largely affect the measure to which neutralization can be influenced. As such, we suspect that species idiosyncrasies could have also played a role in the differences observed. For the above-mentioned reasons, variability in the kinetics of the antibody affinity maturation process, anti-spore activities and species idiosyncrasies in the animals sampled may add to the diversity of the neutralizing ability observed (14).

Another hypothesis for why species have anti-PA antibodies without toxin neutralizing titres (e.g. ENP zebra) or in areas with few anthrax mortalities recorded (e.g. southern KNP) might be due to cross-reaction with closely related antigens to *B. anthracis* PA (88), which needs further investigation. Cross-reactivity will affect the specificity of the technique (PA-ELISA). *Bacillus cereus* biovar *anthracis* and atypical *B. cereus* have been reported to cause anthrax-like infections in humans and animals (92–94). Furthermore, members of *B. cereus sensu stricto* have been reported to be closely related to *B. anthracis* (95). Since TNA quantifies only the neutralizing antibodies in serum, the *B. cereus* isolates with similar *pag* genes may account for the anti-PA positive samples that were negative for TNA (14). Kudu in ENP (44) and zebra in KNP (**Figure 2**) are considered less susceptible (not major hosts) species in these parks. We suggest that their ability to mount neutralizing immune responses against the toxin could be, to an extent, responsible for their protection (89, 90, 96). This hypothesis is based on laboratory studies that reported LT neutralizing antibodies post-vaccination correlated with survival rates in rabbits (*Oryctolagus cuniculus*) (90, 97), guinea pigs (*Cavia porcellus*) (96) and mice (*Mus musculus*) (89).

### Association Between Anti-PA and TN Antibodies

Some studies have demonstrated a correlation between anti-PA antibody titres and toxin neutralizing titres (23, 98). Ndumnego, et al. (23), quantified the anti-PA IgG titres and reported a high correlation with neutralizing antibodies in vaccinated goats (*Capra aegagrus hircus*). Parreiras, et al. (98), compared anti-PA ELISA and TNA in mice vaccinated with PA. In our study, a significant positive correlation was found between the anti-PA ELISA antibody response (SP) and the NT_50_ in animals that naturally acquired the antigen, despite differences between zebra in ENP and kudu in KNP. Although it was seen that anti-PA immune response had an effect on toxin neutralization status in kudu, this was not true in zebra. This result was largely influenced by the zebra in ENP as only a few showed neutralization. However, the correlation observed was expected as neutralizing antibodies are subsets (functional) of the total anti-PA IgG antibodies (14). Not all seropositive animals, based on anti-PA ELISA, showed neutralizing activities, but most animals with neutralizing activities had a high anti-PA titre. Most studies previously conducted were controlled laboratory studies, with animals vaccinated with a predetermined dose and at planned frequencies, which allow for the production of antibodies with high affinity. This is in contrast to this study, where animals were free-roaming and, as such, they encounter pathogen at varying doses and frequencies. This study further confirms the presence of the *B. anthracis* LT antibodies in animal sera.

## Conclusions

Results of this study suggest that immune responses against multi-host pathogens are influenced by several factors (environment, species idiosyncrasies, frequency of exposure, exposure dose), which can be missed from a narrow focus of a single system or species. In this study, the host species from both parks varied in their exposure to *B. anthracis* and immune response to its LT. These patterns may be due to environmental differences between these systems and how they relate to host behavior, which may lead to variation in the frequency of exposure and dose and, in turn, a corresponding immunological trade-off between exposure and tolerance (or resistance) to the anthrax LT. Furthermore, this study revealed that animals in both regions of the parks are exposed to anthrax spores in the environment, which in some cases (e.g., KNP zebra) was inconsistent with anthrax mortality data. As such, our study provides valuable insight into the mechanisms driving variation in anthrax dynamics observed in these parks, with implications for anthrax variation globally.

## Recommendations for Future Research

Future studies examining the role of environmental conditions such as landscape, rainfall, and forage availability on host behavior are needed to establish mechanisms behind the variation in the exposure status of a given host species across locations. Secondly, because of the varying reports in the binding ability of commercially available conjugates, we recommend the development of species-specific conjugates to overcome this limitation. Thirdly, we recommend increased surveillance effort, especially in the “low-incidence” areas, to improve the quality of data currently available. We also recommend that investigation into the role of anthrax risky behaviors or other mechanical vectors in the transmission of *B. anthracis* is needed in ENP to allow comparison to KNP. Further work could investigate the detection of *B. anthracis* in the high versus low incidence regions of these parks as well as the detection of closely related *B. cereus* species in the parks. Future studies could also investigate how exposure frequency and dose affect the correlation between anti-PA antibodies and NT_50_.

## Data Availability Statement

The raw data supporting the conclusions of this article will be made available by the authors, without undue reservation.

## Ethics Statement

The animal study was reviewed and approved by University of Pretoria Research Ethics Committee, Animal Ethics Committee (REC 041-19), Department of Agriculture, Forestry and Fisheries (DAFF) in South Africa (Ref 12/1/1/18) in South Africa, University at Albany’s International Animal Care and Use Committee, approval numbers: 16-016, 18-013, 18-014, 18-015, 20-001, Namibian National Commission on Research, Science and Technology (authorization 2017070704) and the Ministry of Environment, Forestry and Tourism, Namibia.

## Author Contributions

SO, HH, and WT conceived the ideas of the study. SO, HH, WT, and PK designed the study. SO, AxH, OS, ED, Y-HH, and AyH collected the data. SO, JC and HH designed the methodology. SO, CB, and Y-HH analyzed the data. SO and HH wrote the first draft of the manuscript. All authors contributed significantly to manuscript revision, read, and gave approval for publication.

## Funding

This work was supported by NSF Division of Environmental Biology (DEB-1816161/DEB-2106221) to WT, PK, and HH.

## Author Disclaimer

Any use of trade, product, or firm names is for descriptive purposes only and does not imply endorsement by the U.S. Government.

## Conflict of Interest

The authors declare that the research was conducted in the absence of any commercial or financial relationships that could be construed as a potential conflict of interest.

## Publisher’s Note

All claims expressed in this article are solely those of the authors and do not necessarily represent those of their affiliated organizations, or those of the publisher, the editors and the reviewers. Any product that may be evaluated in this article, or claim that may be made by its manufacturer, is not guaranteed or endorsed by the publisher.
