# Supplementary material for: Immunological Evidence of Variation in Exposure and Immune Response to Bacillus anthracis in Herbivores of Kruger and Etosha National Parks
Source: Front Immunol. 2022 Feb 14;13:814031. doi: 10.3389/fimmu.2022.814031 (PMC8882864; doi:10.3389/fimmu.2022.814031)
Supplement: Supplementary file 1 [file DataSheet_1.docx]

Supplementary Material

# Supplementary Data

Supplementary Methodology: Briefly, a pooled sera for each species (impala and kudu) was coated at a dilution of 1:2000 in coating buffer (bicarbonate buffer) per well and incubate overnight at 4^o^C. This was followed by a blocking step where coated plates were blocked with the blocking buffer (200 µL) containing PBST and 5% skimmed milk powder (PBSTM) and then incubated at room temperature for 1 hour. The three commercially available conjugates were tested against kudu (*Tragelaphus* *strepsiceros*) and impala (*Aepyceros* *melampus*) each species. Each conjugate was added to 12 wells of each plate for each species at a dilution of 1:10000. The plates were incubated at room temperature for 30 minutes. Subsequently, the plates were washed after which the ABTS substrate (2,2'-Azinobis [3-ethylbenzothiazoline-6-sulfonic acid]-diammonium salt; Thermo Scientific 1-Step ABTS, USA) was added and allowed in the dark for the colour to develop for 45 minutes. The absorbance was read at 405 nm using a Biotek Powerwave XS2 reader (USA). A one-way ANOVA with a Tukey’s test was performed to determine if there was a statistically significant difference between conjugates. Results are shown in Supplementary Table S2 and Supplementary Figure 1.

# Supplementary Figures and Tables

**Supplementary Table S1:** Mortality data from Kruger National Park (KNP), South Africa, and Etosha National Park (ENP) in Namibia showing the distribution of carcass detection and anthrax mortality. Data for KNP ranged from 1990-2015 and for ENP, from 1996-2015 and the species of study included greater kudu (*Tragelaphus strepsiceros*), plains zebra (*Equus quagga*), impala (*Aepyceros melampus*), and blue wildebeest (*Connochaetes* *taurinus*) and the count (n) for the mortality.

| **Park** | **Part** | **Species** | **Cause** | **n** |
| --- | --- | --- | --- | --- |
| ENP | Central | Kudu | Others | 3 |
| ENP | Central | Others | Others | 299 |
| ENP | Central | Wildebeest | Others | 34 |
| ENP | Central | Zebra | Others | 161 |
| ENP | Central | Others | Anthrax | 120 |
| ENP | Central | Wildebeest | Anthrax | 58 |
| ENP | Central | Zebra | Anthrax | 457 |
| ENP | West | Kudu | Others | 9 |
| ENP | West | Others | Others | 94 |
| ENP | West | Wildebeest | Others | 2 |
| ENP | West | Zebra | Others | 6 |
| ENP | West | Kudu | Anthrax | 1 |
| ENP | West | Others | Anthrax | 6 |
| ENP | West | Zebra | Anthrax | 8 |
| ENP | East | Kudu | Others | 4 |
| ENP | East | Others | Others | 142 |
| ENP | East | Wildebeest | Others | 1 |
| ENP | East | Zebra | Others | 20 |
| ENP | East | Kudu | Anthrax | 1 |
| ENP | East | Others | Anthrax | 10 |
| ENP | East | Wildebeest | Anthrax | 24 |
| ENP | East | Zebra | Anthrax | 17 |
| KNP | North | Impala | Others | 796 |
| KNP | North | Kudu | Others | 160 |
| KNP | North | Others | Others | 1221 |
| KNP | North | Zebra | Others | 211 |
| KNP | North | Impala | Anthrax | 647 |
| KNP | North | Kudu | Anthrax | 1037 |
| KNP | North | Others | Anthrax | 1136 |
| KNP | North | Zebra | Anthrax | 85 |
| KNP | Central | Impala | Others | 94 |
| KNP | Central | Kudu | Others | 22 |
| KNP | Central | Others | Others | 305 |
| KNP | Central | Zebra | Others | 32 |
| KNP | Central | Impala | Anthrax | 1 |
| KNP | Central | Kudu | Anthrax | 16 |
| KNP | Central | Others | Anthrax | 36 |
| KNP | Central | Zebra | Anthrax | 0 |
| KNP | South | Impala | Others | 80 |
| KNP | South | Kudu | Others | 20 |
| KNP | South | Others | Others | 80 |
| KNP | South | Zebra | Others | 5 |

All other species both for anthrax mortality and other causes of death were categorized as “others” (21 different species for anthrax mortality and 57 for other mortality for KNP and 6 different species for anthrax mortality and 27 species for other mortalities in ENP). Mortality data was acquired from Skukuza Veterinary Services and Etosha Ecological Institute.

**Supplementary Table S2:** Optical Density (OD) values of each conjugate against each species at a dilution of 1:20000 for kudu (*Tragelaphus* *strepsiceros*) and impala (*Aepyceros* *melampus*)

| **Species** | **Protein_A** | **Protein_G** | **Protein AG** |
| --- | --- | --- | --- |
| Impala | 0.25 | 3.982 | 2.912 |
| Impala | 0.589 | 3.982 | 2.494 |
| Impala | 0.611 | 3.971 | 2.348 |
| Impala | 0.594 | 3.977 | 2.42 |
| Impala | 0.226 | 3.791 | 2.744 |
| Impala | 0.602 | 3.789 | 2.455 |
| Impala | 0.622 | 3.799 | 2.393 |
| Impala | 0.608 | 3.858 | 2.367 |
| Impala | 0.27 | 3.887 | 2.568 |
| Impala | 0.585 | 3.896 | 2.162 |
| Impala | 0.6 | 3.885 | 2.134 |
| Impala | 0.628 | 3.796 | 2.211 |
| Kudu | 1.392 | 3.872 | 2.657 |
| Kudu | 1.737 | 3.683 | 2.821 |
| Kudu | 1.775 | 3.911 | 2.795 |
| Kudu | 1.769 | 3.872 | 2.783 |
| Kudu | 1.257 | 3.728 | 2.509 |
| Kudu | 1.764 | 3.837 | 2.845 |
| Kudu | 1.837 | 3.82 | 2.861 |
| Kudu | 1.795 | 3.863 | 2.86 |
| Kudu | 1.233 | 3.892 | 2.197 |
| Kudu | 1.755 | 3.677 | 2.721 |
| Kudu | 1.833 | 3.793 | 2.53 |
| Kudu | 1.843 | 3.781 | 2.414 |

**Supplementary Table S3.** A generalised linear model (Gaussian distribution) for the significance of anti-protective antigen (PA) antibodies. Optical density (OD) values were measured using an anti-PA ELISA, and mean sample to positive (SP) ratios were estimated for all sampled animals in a given location. SD is the standard deviation. Areas of high and low incidence in each park (ENP = Etosha National Park and KNP = Kruger National Park) are shown in Figure 1. The species of study included greater kudu (*Tragelaphus strepsiceros*), plains zebra (*Equus quagga*), impala (*Aepyceros melampus*), and blue wildebeest (*Connochaetes* *taurinus*). Separate multivariable models were performed for kudu and zebra

| **Variable** | **Category** | **No. of animals sampled** | **Mean SP ± SD** | **Coefficient** | ***p*-value** |
| --- | --- | --- | --- | --- | --- |
| **Kudu** |  |  |  |  |  |
| **National park** | ENP | 40 | 0.65 ± 0.07 |  |  |
|  | KNP | 37 | 1.28 ± 0.12 | 1.33 | 0.047 |
| **Location** | Low incidence | 38 | 0.85 ± 0.11 |  |  |
|  | High incidence | 39 | 1.06 ± 0.11 | 0.89 | 0.41 |
| **TNA status** | Negative | 33 | 0.79 ± 0.13 |  |  |
|  | Positive | 44 | 1.08 ± 0.09 | 1.35 | 0.004 |
| **Interaction: Park*location status (KNP*incidence area)** |  |  |  | 1.64 | 0.015 |
| **Zebra** |  |  |  |  |  |
| **National park** | KNP | 40 | 0.53 ± 0.06 |  |  |
|  | ENP | 40 | 0.69 ± 0.08 | 1.33 | 0.034 |
| **Incidence status** | Low incidence | 40 | 0.55 ± 0.08 |  |  |
|  | High Incidence | 40 | 0.67 ± 0.07 | 1.12 | 0.29 |
| **TNA status** | Negative | 47 | 0.60 ± 0.08 |  |  |
|  | Positive | 33 | 0.62 ± 0.07 | 1.22 | 0.15 |
|  |  |  |  |  |  |
| **Wildebeest** | ENP (High incidence) | 20 | 0.32 ± 0.05 | NA | NA |
| **Impala** | KNP (Low incidence) | 20 | 0.31 ± 0.04 | NA | NA |

**Supplementary Table S4.** A generalised linear model (Gaussian distribution) for the significance of *Bacillus anthracis* lethal toxin (LT) neutralisation scores from wildlife species sampled in two national parks in southern Africa. The neutralisation titre 50 (NT_50_) is the highest titre that protected 50% of mouse macrophage cells. ELISA is the enzyme linked immunosorbent assay; SD is the standard deviation, and mean sample to positive (SP) ratios were estimated for all sampled animals in a given location. Areas of high and low incidence in each park (ENP = Etosha National Park and KNP = Kruger National Park) are shown in Figure 1. Serum samples were collected from kudus (*Tragelaphus strepsiceros*), and zebras (*Equus quagga*) in Kruger National Park (KNP) in South Africa and Etosha National Park (ENP) in Namibia (see Figure 1 for parks and sub-locations).

| **Variable** | **Category** | **No. of animals sampled** | **Mean NT_50_ ± SD** | **Exp (coefficient)** | ***p*-value** |
| --- | --- | --- | --- | --- | --- |
|  |  |  |  |  |  |
| **Host species** | Zebra | 33 | 70.85 ± 7.8 |  |  |
|  | Kudu | 44 | 91.25 ± 11.6 | 0.68 | <0.0001 |
| **National park** | KNP | 47 | 71.07 ± 7.3 |  |  |
|  | ENP | 30 | 100.43 ± 15.0 | 0.83 | 0.11 |
| **Sub-location^a^** | High incidence | 41 | 81.72 ± 9.4 |  |  |
|  | Low incidence | 36 | 83.41 ± 12.0 | NA | NA |
| **Host species x National park** |  |  |  | 1.96 | <0.0001 |
| **ELISA SPs (Log)** |  |  |  | 1.76 | <0.0001 |

^a^ Sub-location was not included in the final Gaussian model because the variable was not significant

## Supplementary Figures


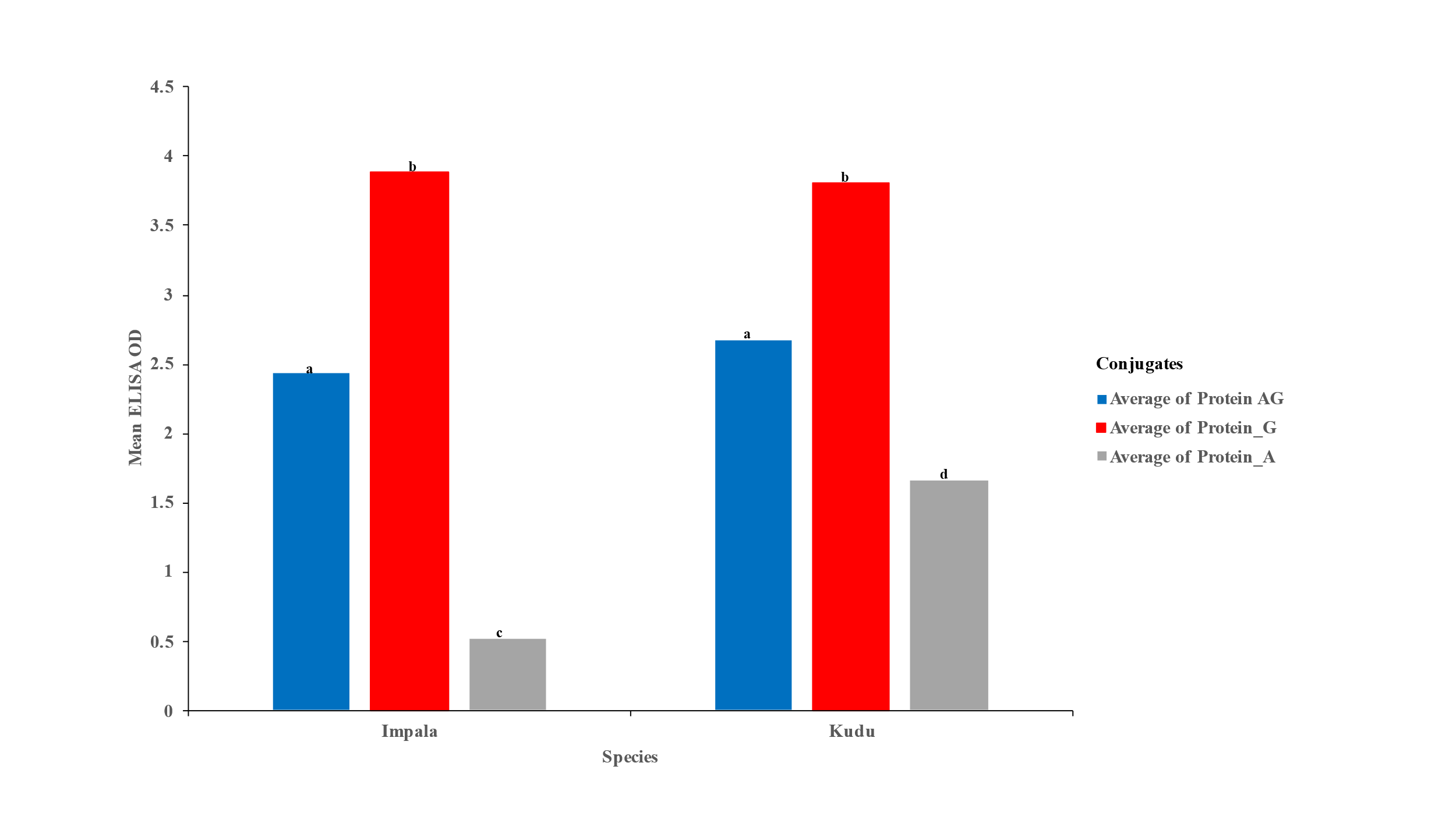


**Supplementary Figure 1.** Bar graph showing the mean optical densities (OD) of three different protein conjugates for kudu (*Tragelaphus strepsiceros*) and impala (*Aepyceros melampus*). Different lower-case letters above each bar indicate statistically significant differences (p<0.05) between the different conjugates across the two species.
